# Toxic Effects of Copper Fungicides on the Development and Behavior of Zebrafish in Early-Life Stages

**DOI:** 10.3390/nano13192629

**Published:** 2023-09-23

**Authors:** Fei Gao, Zitong Yuan, Lingling Zhang, Yiyuan Peng, Kun Qian, Mingqi Zheng

**Affiliations:** 1 College of Science, China Agricultural University, Beijing 100193, China; s20173101495@cau.edu.cn (F.G.); zhangll@cau.edu.cn (L.Z.); 2 College of Plant Protection, Southwest University, Chongqing 400715, China; yuanzitong@email.swu.edu.cn (Z.Y.); yuan6893124@email.swu.edu.cn (Y.P.); qk1982@swu.edu.cn (K.Q.)

**Keywords:** zebrafish, early-life stages, copper fungicides, developmental and behavioral toxicity

## Abstract

Copper-based fungicides have been used to control various plant diseases for more than one hundred years and play very important roles in agriculture. Accumulation of copper in freshwater and environment pose severe threats to human health and the environment. The current study evaluated the developmental and behavioral toxicity of PEG@Cu NCs (copper nanoclusters), Kocide^®^ 3000 (copper hydroxide), and Cu(CH_3_COO)_2_ (copper acetate) to zebrafish in early-life stages. The developmental toxicity was evaluated according to the parameters of mortality, hatching rate, autonomous movement and heartbeat of embryos, and body length of larvae. The 9 dpf (days postfertilization)-LC_50_ (50% lethal concentration) of embryonic mortality was 0.077, 0.174 or 0.088 mg/L, and the 9 dpf-EC_50_ (effective concentration of 50% embryos hatching) of hatching rate was 0.079 mg/L, 0.21 mg/L and 0.092 mg/L when the embryos were exposed to PEG@Cu NCs, Kocide^®^ 3000 or Cu(CH_3_COO)_2_, respectively. Kocide^®^ 3000 and Cu(CH_3_COO)_2_ obviously decreased the spontaneous movements, while PEG@Cu NCs had no adverse effects on that of embryos. The reduced heartbeat can return to normal after exposure to PEG@Cu NCs for 96 h, while it cannot recover from Kocide^®^ 3000. In addition, Kocide^®^ 3000 (≥0.2 mg/L), PEG@Cu NCs and Cu(CH_3_COO)_2_ with 0.05 mg/L or higher concentration exhibited obvious behavioral toxicity to zebrafish larvae according to the parameters of movement distance, average velocity, absolute sinuosity, absolute turn angle and absolute angular velocity.

## 1. Introduction

Copper exists naturally in a variety of mineral forms, and is widely used in industry, agriculture, cosmetics and food processing, etc. [1]. Copper is an essential element for all forms of life and plays very important roles in many physiological and biochemical processes of living organisms. In addition, it also serves as a cofactor for several enzymes [2,3]. However, copper can cause hydromineral regulatory malfunction, stressing or killing organisms, when the copper concentrations exceed the nutritional needs [4]. Disruptions in the homeostatic mechanisms of copper metabolism in brain are associated with human neurodegenerative disorders such as Menkes disease, Wilson’s disease, Alzheimer’s disease, Parkinson’s disease, and amyotrophic lateral sclerosis [5,6]. In addition, copper displayed adverse effects on various aquatic organisms, including fish, algae, *Daphnia*, etc. Copper affected the normal growth and survival of fish by interfering in the nervous system, innate immune system and antioxidant system [7,8]. It also affected the reproduction and survival ability of *Daphnia* species [9,10]. In addition, copper can reduce photosynthetic efficiency, induce oxidative stress, and inhibit the growth of algae [11,12].

Copper was one of the first elements used as a plant fungicide. It can be traced back to the famous origin of Bordeaux mixture, containing a mixture of copper sulfate and lime, which could effectively control the destructive diseases of downy mildew in France vineyards [13]. At present, a large number of copper fungicides are used for controlling diseases such as citrus canker (*Xanthomonas citri* subsp. *Citri*), angular leaf spot of cucumber (*Pseudomonas syringae* pv. *Lachrymans*), and downy mildew disease of grape (*Plasmopara viticola*) and cucumber (*Pseudoperonospora cubensis*) all over the world, including China [14,15]. These copper fungicides include copper sulfate, copper hydroxide, copper oxychloride and copper acetate, etc. [13,15]. With the application of nanotechnology in pesticide processing, Cu-based chemicals are one of the most common forms of nanopesticides due to their excellent properties and low cost [16]. Compared with traditional pesticides, nanopesticides can improve bioavailability, enhance solubility, delay the degradation rate of active ingredients, control the release rate of active ingredients, etc. [17]. However, nanopesticides may also have unexpected consequences for nontarget organisms and the environment due to the inherent characteristics of the nanoparticles and the release of unknown chemical entities into the environment [17,18].

Copper usually enters the environment with aqueous discharges, such as urban wastewater, industrial and mine effluent and agriculture runoff, etc. [1,19]. A total of approximately 8 kt of copper per annum (ktpa) is estimated to enter freshwater in the European Union (EU), while approximately 1.8 ktpa is estimated to enter freshwater by the way of agriculture [1]. It has been reported that copper concentration was about 10 mg/L and 100 mg/L in aquatic systems near cities and mining areas, respectively [19]. Copper in an aquatic environment can profoundly influence both human and environmental health. To date, there are more than 400 copper-based fungicides registered for controlling a large number of plant diseases [15]. In this study, the toxic effects of fungicides of Kocide^®^ 3000 (copper hydroxide), Cu(CH_3_COO)_2_ (copper acetate), and copper nanoclusters of PEG@Cu NCs (copper nanoclusters) on the development and behavior of zebrafish in early-life stages were studied.

## 2. Materials and Methods

### 2.1. Chemicals and Reagents

PEG@Cu NCs, a copper nanocluster pesticide, was provided by Professor Kun Qian from Southwest University of China (Chongqing, China). Kocide^®^ 3000 (46% copper hydroxide water-dispersible granule) is a commercial pesticide produced by DuPont Company (Wilmington, DE, USA), and 98% copper acetate anhydrous was purchased from Shanghai Macklin Biochemical Co., Ltd (Shanghai, China). Reconstituted water of pH 7.5 ± 0.5 was prepared according to the guideline of ISO-7346-2 with minor adjustments, which mainly contained 294 mg/L CaCl_2_ · 2H_2_O, 106.5 mg/L NaHCO_3_, 60 mg/L MgSO_4_ and 6 mg/L KCl [20].

### 2.2. Characterization of PEG@Cu NCs and Kocide^®^ 3000

The shape, particle size of PEG@Cu NCs and Kocide^®^ 3000 was measured by transmission electron microscopy (TEM) (JEOL JEM-F200, Tokyo, Japan) [21]. The hydrodynamic particle size of Kocide^®^ 3000 was measured by a nanoparticle-size and zeta potential analyzer (Malvern Zetasizer Nano ZS90, Worcestershire, UK).

### 2.3. Maintenance of Zebrafish and Embryo Collection

Adult zebrafish (wild type, AB strain) were purchased from Beijing Hongda Gaofeng Aquarium Department, China. The male and female zebrafish were separately raised in the circulating filtration system (Beijing ESEN Technology Development Co., Ltd.; Beijing, China) under conditions of 26 ± 1 °C and photoperiod of 14 h/10 h (light/dark). Adult zebrafish were fed with live brine shrimp three times a day.

The night before the test, the male and female parent fish in a ratio of 1:1 were put into the spawning box, which was separated by a baffle and covered with black cloth for dark treatment. The fertilized embryos were collected and washed twice in reconstituted water the next morning. The normal and healthy embryos within 2 h postfertilization (hpf) were selected for subsequent tests.

### 2.4. Acute Toxicity Test of Copper Fungicides on Embryos

Acute toxicity testing of embryos was carried out according to the OECD guideline 236 [22] with modifications. The final concentration of test solutions was 0.0125, 0.05, 0.2, 0.8, and 3.2 mg/L for PEG@Cu NCs, 0.23, 0.26, 0.3, 0.35, and 0.4 mg/L for Cu(CH_3_COO)_2_, and 0.25, 0.5, 1, and 2, 4 mg/L for Kocide^®^ 3000.

A total of 15 healthy embryos within 2 hpf were selected and placed in 40 mL test solutions with different concentrations. The reconstituted water was used as control. Three replicates were set for each test concentration and control. Test embryos were raised in an incubator in conditions of 26 ± 1 °C and photoperiod of 14 h/10 h (light/dark) for 96 h. The dead embryos were recorded and removed every 24 h during test periods. All procedures in this test complied with Chinese legislation and were approved by the Independent Animal Ethics Committee of China Agricultural University.

### 2.5. Developmental Toxicity Test of Zebrafish in Early-Life Stages

The experiment was conducted according to the OECD guideline 212 [23] with modifications. A series concentration of 0.0125, 0.05, 0.2, 0.8 and 3.2 mg/L was set for PEG@Cu NCs, Cu(CH_3_COO)_2_ or Kocide^®^ 3000. A total of 15 healthy embryos were selected and immersed in 50 mL test solution with different concentrations. The reconstituted water was used as control. Test embryos were raised in an incubator in conditions of 26 ± 1 °C and photoperiod of 14 h/10 h (light/dark) for 9 days. The parameters of mortality and hatching rate, autonomous movement, heartbeat, locomotor behavior, body length and deformity were observed and recorded at different times during the test periods. The methods for locomotor behavior are described in Section 2.6.

### 2.6. Locomotor Behavior of Zebrafish in Early-Life Stages

At 7 days postfertilization (dpf), the larvae without morphological abnormalities were selected and transferred into 24-well plates with one larva in 2 mL solution per well. At 8 dpf, all larvae were removed from incubators and acclimated at room temperature for 2 h. Then, the movement behavior of individual larvae was continuously recorded for 10 min in dark conditions using a DanioVision system (Noldus, Wageningen, The Netherlands) after acclimation in an observation chamber for 10 min. The total distance traveled, average velocity, absolute sinuosity, absolute turn angle, and absolute angular velocity were analyzed using EthoVision XT15 software (Noldus, Wageningen, The Netherlands). Tests were carried out in triplicate (*n* = 24) for each concentration.

### 2.7. Statistical Analysis

Significance analysis of means was conducted by one-way analysis of variance (ANOVA).

## 3. Results

### 3.1. Characterization of PEG@Cu NCs and Kocide^®^ 3000

The shape of PEG@Cu NCs is nearly spherical, and the size is approximately 72.73 nm (Figure S1A). In contrast, the shape of Kocide^®^ 3000 particle is irregular, and the average particle size cannot be determined accurately (Figure S1B). The hydrodynamic particle size of Kocide^®^ 3000 was 768.6 ± 148.3 nm, which indicated that Kocide^®^ 3000 is a nanopesticide (Figure S2).

### 3.2. Acute Toxicity Test of Copper Fungicides on Embryos

The results indicated that the 96 hpf-LC_50_ (50% lethal concentration) of PEG@Cu NCs and Cu(CH_3_COO)_2_ for embryos was 0.166 mg/L and 0.276 mg/L, respectively (Table 1 and Table S1). The mortality of embryos was 7.78%, 6.67% and 16.67% when the concentration of Kocide^®^ 3000 was 1.0, 2.0 and 4.0 mg/L, respectively. However, deposits of Kocide^®^ 3000 were observed in 4 mg/L test solutions. Hence, the 96 hpf-LC_50_ of Kocide^®^ 3000 is thought to be more than 2 mg/L.

### 3.3. Developmental Toxicity of Copper Fungicides in Zebrafish in Early-Life Stages

#### 3.3.1. Mortality of Embryo

The copper fungicides exhibited different toxicity to embryos (or larvae). The 9 dpf-LC_50_ of PEG@Cu NCs, Kocide^®^ 3000 and Cu(CH_3_COO)_2_ to embryos was 0.077, 0.174 and 0.088 mg/L, respectively (Table 2). The PEG@Cu NCs and Cu(CH_3_COO)_2_ displayed higher embryo (or larvae) toxicity to zebrafish in early-life stages than Kocide^®^ 3000.

**Figure 1:**
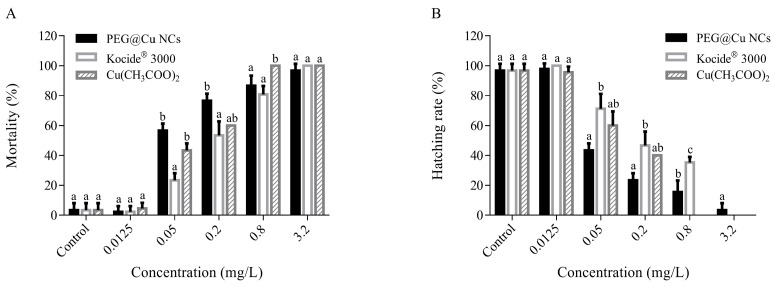
The 9 dpf mortality (**A**) or hatching rate (**B**) of embryos after exposure to PEG@Cu NCs, Kocide^®^ 3000 and Cu(CH_3_COO)_2_ at concentrations of 0.0125, 0.05, 0.2, 0.8 and 3.2 mg/L. The hatching rate is zero, because all embryos died after exposure to 3.2 mg/L Kocide^®^ 3000 or Cu(CH_3_COO)_2_. Values are shown as means ± SD (standard deviation). The mortality and hatching rate with different letter are significantly different (*p* < 0.05) for the same concentration of different fungicides.

The mortality of embryos increased with the enhancement of copper concentration (Figure 1A). The embryo mortality (about 2.22–4.45%) displayed no obvious difference treated with PEG@Cu NCs, Kocide^®^ 3000 and Cu(CH_3_COO)_2_ with concentration of 0.0125 mg/L. At a concentration of 0.05 mg/L, the embryo mortality treated with PEG@Cu NCs and Cu(CH_3_COO)_2_ was significantly higher than Kocide^®^ 3000. However, almost all embryos died after exposure to PEG@Cu NCs, Kocide^®^ 3000 and Cu(CH_3_COO)_2_ at concentration of 3.2 mg/L.

#### 3.3.2. Hatching Rate of Embryos

The 9 dpf-EC_50_ (effective concentration of 50% embryos hatching) of PEG@Cu NCs, Kocide^®^ 3000 and Cu(CH_3_COO)_2_ to embryos was 0.079, 0.21 and 0.092 mg/L, respectively (Table 2), which indicated that the copper fungicides displayed different effects on hatching rate of embryo.

For each copper fungicide, the hatching rate of embryos decreased with enhancement in copper concentration (Figure 1B). The hatching rate of embryos displayed no significant difference after exposure to PEG@Cu NCs, Kocide^®^ 3000 or Cu(CH_3_COO)_2_ at 0.0125 mg/L. At concentrations of 0.05 and 0.2 mg/L, the hatching rate of embryos between the PEG@Cu NCs and Cu(CH_3_COO)_2_ had no difference, but the hatching rate of embryos exposed to PEG@Cu NCs was significantly lower than that of Kocide^®^ 3000. The hatching rate of embryos treated with PEG@Cu NCs was obviously lower than Kocide^®^ 3000, but significantly higher than Cu(CH_3_COO)_2_ at concentration of 0.8 mg/L. When the concentration of PEG@Cu NCs, Kocide^®^ 3000 or Cu(CH_3_COO)_2_ increased to 3.2 mg/L, almost no embryos could hatch.

#### 3.3.3. Autonomous Movement of Embryos

**Figure 2:**
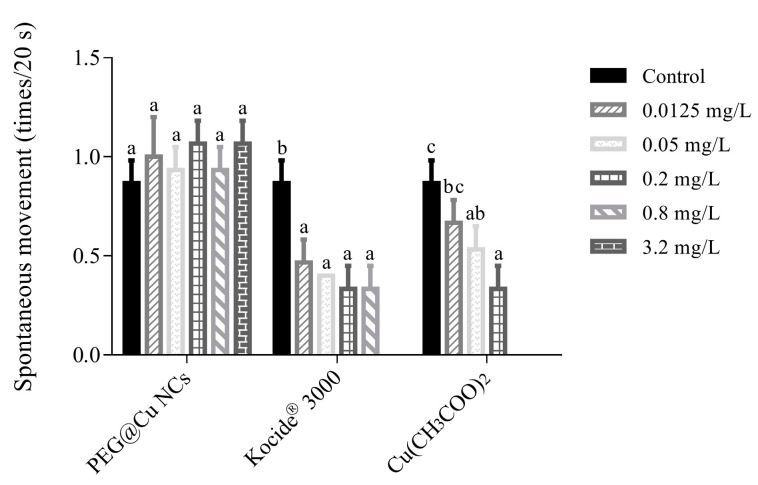
Spontaneous movement of zebrafish embryos after exposure to PEG@Cu NCs, Kocide^®^ 3000 and Cu(CH_3_COO)_2_ for 48 h. Values are shown as means ± SD. Different letters indicate significant differences (*p* < 0.05) for the same fungicide.

The three copper fungicides exhibited different effects on the autonomous movements of embryos (Figure 2). The spontaneous movement of embryos was not affected by PEG@Cu NCs at concentrations of 0.0125–3.2 mg/L. In contrast, Kocide^®^ 3000 (0.0125–0.8 mg/L) and Cu(CH_3_COO)_2_ (0.05–0.2 mg/L) obviously decreased the spontaneous movements of embryos. Almost all the embryos died after exposure to 3.2 mg/L Kocide^®^ 3000 or Cu(CH_3_COO)_2_ at 0.8 mg/L and 3.2 mg/L.

#### 3.3.4. Heartbeat of Zebrafish in Early-Life Stages

**Figure 3:**
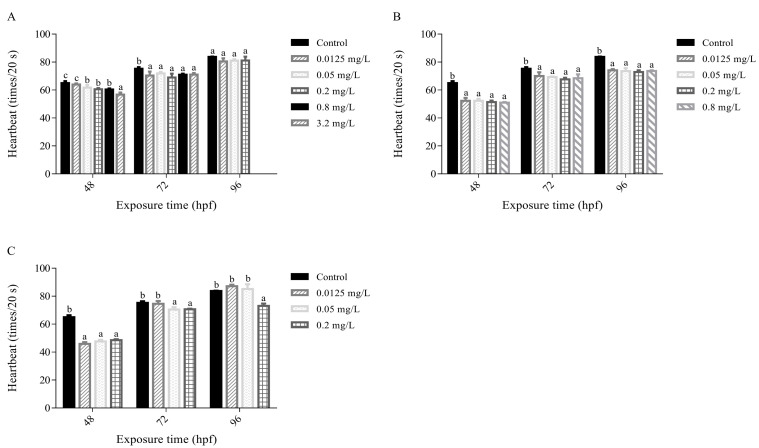
The heartbeat number in 20 s of embryos after exposure to PEG@Cu NCs (**A**), Kocide^®^ 3000 (**B**) and Cu(CH_3_COO)_2_ (**C**) at different times. Values are shown as means ± SD. Different letters indicate significant differences (*p* < 0.05) at the same exposure time.

The copper fungicides exhibited different effects on the heartbeat of zebrafish in early-life stages with change in exposure time and concentrations (Figure 3A–C). In general, the heartbeat of zebrafish in early-life stages treated with PEG@Cu NCs, Kocide^®^ 3000 or Cu(CH_3_COO)_2_ with different concentrations gradually returned with the extension of exposure time within 48–96 h. The heartbeat reduced about 1.5–12.6%, 19.4–21.6% and 25.3–29.3% after exposure to PEG@Cu NCs (0.0125–3.2 mg/L), Kocide^®^ 3000 (0.0125–0.8 mg/L) and Cu(CH_3_COO)_2_ (0.0125–0.2 mg/L) for 48 h, respectively (Figure 3A).

At 72 hpf, the heartbeat of zebrafish in early-life stages treated with PEG@Cu NCs, Kocide^®^ 3000 or Cu(CH_3_COO)_2_ recovered gradually, and the number of heartbeats was about 93.9%, 91.6% and 95.6% of the control, respectively (Figure 3B). As the exposure time increased to 96 h, the heartbeat after treatment with PEG@Cu NCs (0.0125–0.2 mg/L) or Cu(CH_3_COO)_2_ (0.0125–0.05 mg/L) returned to normal. However, the heartbeat after treatment with 0.2 mg/L Cu(CH_3_COO)_2_ reduced greatly. In contrast, heartbeat after treatment with Kocide^®^ 3000 (0.0125–0.8 mg/L) was still inhibited, the number of heartbeats was about 87.0–88.5% of control (Figure 3C).

#### 3.3.5. Body Length of Zebrafish Larvae

**Figure 4:**
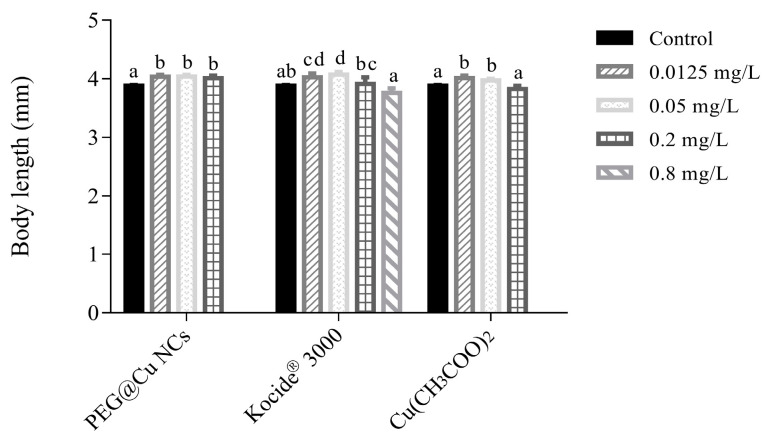
Effects of PEG@Cu NCs, Kocide^®^ 3000 and Cu(CH_3_COO)_2_ on body length of larvae at 216 hpf. Values are shown as means ± SD. Different letters indicate significant differences (*p* < 0.05) for the same copper fungicide.

Collectively, the copper fungicides at low concentrations displayed obvious promotion effects on the growth of zebrafish larvae (Figure 4). The body length increased about 2.6–4.0% after exposure to PEG@Cu NCs at 0.0125–0.02 mg/L compared with control zebrafish larvae. When treated with Kocide^®^ 3000, the body length of zebrafish larvae significantly increased (increased about 3.7–4.9%) at 0.0125–0.05 mg/L, while it returned to normal at 0.2 mg/L and 0.8 mg/L. In contrast, Cu(CH_3_COO)_2_ stimulated growth (2.3–3.4%) at 0.0125 mg/L and 0.05 mg/L, and the body length of zebrafish larvae returned to normal after exposure to 0.2 mg/L Cu(CH_3_COO)_2_.

### 3.4. Behavioral Responses of Zebrafish Larvae

#### 3.4.1. Total Movement Distance and Average Velocity

**Figure 5:**
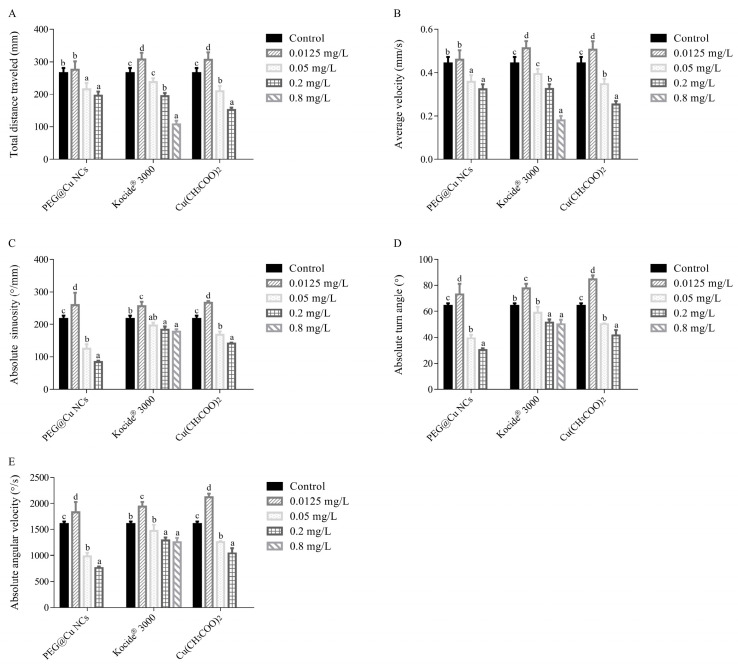
The behavioral parameters of total movement distance (**A**), average velocity (**B**), absolute sinuosity (**C**), absolute turn angle (**D**), and absolute angular velocity (**E**) of zebrafish larvae after exposure to PEG@Cu NCs, Kocide^®^ 3000 and Cu(CH_3_COO)_2_ at different concentrations. Values are shown as means ± SD. Different letters indicate significant differences (*p* < 0.05) for the same copper fungicide.

The total movement distance and average velocity of zebrafish larvae increased obviously after exposure to 0.0125 mg/L of Kocide^®^ 3000 or Cu(CH_3_COO)_2_ compared with that of control larvae. However, the total movement distance and average velocity of zebrafish larvae reduced significantly when the exposure concentration increased to 0.05 mg/L or above (Figure 5A,B).

#### 3.4.2. Absolute Turn Angle

The absolute turn angle of zebrafish larvae increased significantly compared with that of control larvae when treated with 0.0125 mg/L of PEG@Cu NCs, Kocide^®^ 3000 or Cu(CH_3_COO)_2_. However, the absolute turn angle of zebrafish larvae decreased obviously when the exposure concentration increased to 0.05 mg/L or above (except 0.05 mg/L Kocide^®^ 3000). When treated with PEG@Cu NCs, Kocide^®^ 3000 or Cu(CH_3_COO)_2_ at 0.05 mg/L and 0.2 mg/L, the absolute turn angle decreased about 39.0–52.8%, 8.5–20.0%, and 22.0–35.3%, respectively (Figure 5C).

#### 3.4.3. Absolute Sinuosity and Absolute Angular Velocity

The absolute sinuosity and absolute angular velocity of zebrafish larvae increased obviously after exposure to 0.0125 mg/L PEG@Cu NCs, Kocide^®^ 3000 or Cu(CH_3_COO)_2_. The absolute sinuosity and absolute angular velocity of zebrafish larvae decreased significantly after exposure to PEG@Cu NCs or Cu(CH_3_COO)_2_ at concentrations of 0.05–0.2 mg/L. The decrease was 42.0–61.2% and 38.9–52.8% for PEG@Cu NCs, 22.2–35.1% and 21.9–35.3% for Cu(CH_3_COO)_2_. In contrast, Kocide^®^ 3000 reduced the absolute sinuosity and absolute angular velocity of zebrafish larvae only at 0.2 mg/L and 0.8 mg/L (Figure 5D,E).

## 4. Discussion

The early-life stages of zebrafish mainly included embryos and larvae, which were widely used for the studies on aquatic toxicity as experimental models due to sensitivity to environment pollutants, including pesticides. In this study, the adverse effects of PEG@Cu NCs, Kocide^®^ 3000 and Cu(CH_3_COO)_2_, which are copper-based fungicides, on the development and behavior of zebrafish in early-life stages were evaluated.

### 4.1. Developmental Toxicity of Copper Fungicides to Zebrafish in Early-Life Stages

The mortality, hatching rate, autonomous movement, heartbeat, and body length of zebrafish in early-life stages were used for evaluating the development toxicity of three copper fungicides in this study.

Hatching is very important in the development of zebrafish embryos and is considered a critical point for assessing the effects of toxic substances on fish in early stages [24]. In this study, the PEG@Cu NCs, Kocide^®^ 3000 and Cu(CH_3_COO)_2_ exhibited obvious adverse effects on the hatching rate of embryos. The hatching rate of embryos decreased significantly as the concentration of the copper fungicides increased to 0.05 mg/L, and almost all embryos died at 3.2 mg/L (Figure 1). Similar phenomena were observed in other experiments. For example, the hatching rate of zebrafish embryos (<1 hpf) decreased significantly after exposure to 0.053 mg/L or higher concentrations of Cu^2+^ (CuSO_4_·H_2_O) for 72 h [25]. A decrease in hatching rate was observed at 48 hpf, when zebrafish embryos (4 hpf) were exposed to Cu(OH)_2_ nanopesticide (CNPE) (Kocide 3000) at 4 mg/L or higher concentration [26]. The mechanisms involved in the hatching delay caused by exposure to pollutants are not clear, and it has been proposed that the decrease in embryo motility, changes in the levels of hatching enzymes or alteration of oxygen uptake by the embryos can contribute to hatching inhibition [27,28]. In addition, the PEG@Cu NCs displayed similar toxicity to Cu(CH_3_COO)_2_ and higher toxicity than Kocide^®^ 3000 at concentrations of 0.05 mg/L and 0.2 mg/L according to the hatching rate of embryos in this study. This may be caused by the characteristics of copper nanoparticles. The diameter of the chorionic membrane is approximately 0.5–0.7 µm, so copper nanoparticles are more likely to enter the embryonic chorionic membrane [29]. Nanoparticles can also accumulate on the chorionic membrane, blocking the pores and reducing oxygen passage, thus delaying hatching [30,31]. In addition, Cu^2+^ delay or impaired hatching of fish embryos occurred after inhibiting hatching enzyme activity, inducing reactive oxygen species (ROX) and downregulating wingless-type MMTV integration site family (Wnt) signaling [27,28,32].

The frequency of embryonic voluntary movements is associated with neurodevelopment and is one of the important biomarkers in neurodevelopmental toxicity studies [33,34]. The results on voluntary movement indicated that Kocide^®^ 3000 (0.0125–0.8 mg/L) and Cu(CH_3_COO)_2_ (0.05–0.2 mg/L) obviously decreased the spontaneous movements of embryos. This demonstrated that Kocide^®^ 3000 and Cu(CH_3_COO)_2_ had neurotoxicity to the embryo. However, PEG@Cu NCs at concentrations of 0.0125–3.2 mg/L had no obvious effects on spontaneous movements compared with that of control. Other studies also confirmed that oxine copper and Kocide^®^ 3000 nanopesticide reduced the spontaneous movement of embryos greatly [26,34]. This spontaneous movement behavior is considered to be related to the electric coupling network of a subset of spinal neurons [35,36]. Hence, the copper fungicides may affect the autonomous movement of embryos by disrupting the electrical coupling network of related neurons in the spinal cord.

The heart is one of the first organs formed during the embryonic development of zebrafish. Hence, heartbeat is an important parameter for evaluating cardiac function and toxicity of pesticides to zebrafish embryos [37,38]. The results in this study indicated the three copper fungicides displayed different effects on the heartbeat of zebrafish in early-life stages. The heartbeat could recover to normal at 96 hpf when treated with PEG@Cu NCs at all concentrations (0.0125–0.8 mg/L). In addition, the heartbeat could recover to normal after exposure to Cu(CH_3_COO)_2_ only at 0.0125 and 0.05 mg/L. However, the heartbeat could not recover to normal after exposure to Kocide^®^ 3000 under any concentration (0.0125–0.8 mg/L). The adverse effects of copper on the heartbeat were confirmed in various experiments. For example, CuSO_4_·5H_2_O at concentration of 327 μg/L and 464 μg/L significantly increased heartbeat of embryos at 28 hpf [25], while Cu(OH)_2_ nanopesticide (CNPE) (Kocide 3000) (at 4 mg/L or higher), CuO NPs (1 mg/L or higher), and oxine copper (10 μg/L) significantly decreased heartbeat of zebrafish embryos [26,34,37]

Body length is also one of the important parameters measuring zebrafish growth. The results demonstrated that PEG@Cu NCs, Kocide^®^ 3000, and Cu(CH_3_COO)_2_ obviously promoted the growth of larvae at 0.0125 and 0.05 mg/L, and did not exhibit inhibition to body length of larvae at 0.2 mg/L or 0.8 mg/L (Figure 4). In contrast, a lot of studies confirmed that copper can inhibit the body growth of zebrafish at different times [25,26,32,39]. Whether copper, as an essential trace element in organisms, promotes or inhibits growth may be directly related to its concentration.

### 4.2. Behavioral Toxicity of Copper Fungicides to Zebrafish in Early-Life Stages

Behavioral changes have been proven to be a sensitive and important endpoint for detecting contaminant-induced neurological damage [40,41]. In the present study, the parameters of total distance traveled, average velocity, absolute sinuosity, absolute turn angle and absolute angular velocity were used for evaluating the behavioral toxicity of three copper fungicides to zebrafish in early-life stages.

The results indicated that the parameters of total distance traveled, average velocity, absolute sinuosity, absolute turn angle and absolute angular velocity obviously reduced when the zebrafish were exposed to Kocide^®^ 3000 (≥0.2 mg/L), PEG@Cu NCs and Cu(CH_3_COO)_2_ at 0.05 mg/L or higher concentrations (Figure 5). This suggested that the swimming behavior of the larvae was impaired obviously by the copper fungicides. Reference [39] reported that Cu (CuSO_4_·5H_2_O) at 125 μg/L reduced the mean velocity, total distance traveled and absolute turn angle of larvae. The 50 mg/L copper oxide nanoparticles (CuO NPs) reduced obviously the total movement distance, velocity, and angular velocity of larvae [42]. Oxine copper (10, 20, 40 μg/L) decreased the total distance, average speed and movement time of larvae [34]. The disorder in behavioral patterns may be associated with abnormalities in musculoskeletal structure and changes in the nervous system [39,41,43].

## 5. Conclusions

The results in this study indicated that copper fungicides (Kocide^®^ 3000, PEG@Cu NCs and Cu(CH_3_COO)_2_) exhibited developmental and behavioral toxicity to embryos or zebrafish in early-life stages. In contrast, the adverse effects of PEG@Cu NCs on the growth and development of zebrafish in early-life stages seems to be less than Kocide^®^ 3000 and Cu(CH_3_COO)_2_ according to the parameters of spontaneous movements of embryo, heartbeat and body length. However, the mechanisms underlying the developmental and behavioral toxicity are not clear and need to be thoroughly investigated in subsequent experiments.

## Figures and Tables

**Table 1:** The acute toxicity of PEG@Cu NCs, Kocide^®^ 3000 and Cu(CH_3_COO)_2_ for zebrafish embryos.

Fungicides	96 hpf—LC_50_ (mg/L)	95% Confidence Limit (mg/L)	R^2^
PEG@Cu NCs	0.166	0.086–0.312	0.96
Kocide^®^ 3000	>2.0	-	-
Cu(CH_3_COO)_2_	0.276	0.256–0.296	0.96

Note: hpf = hours postfertilization; LC_50_ = 50% lethal concentration.

**Table 2:** The 9 dpf mortality and hatching rate for zebrafish embryos exposed to PEG@Cu NCs, Kocide^®^ 3000 and Cu(CH_3_COO)_2_.

Fungicides	Mortality			Hatching Rate		
	9 dpf—LC_50_ (mg/L)	95% CL (mg/L)	R^2^	9 dpf—EC_50_ (mg/L)	95% CL (mg/L)	R^2^
PEG@Cu NCs	0.077	0.005–0.343	0.87	0.079	0.003–0.396	0.87
Kocide^®^ 3000	0.174	0.107–0.280	0.98	0.210	0.039–1.160	0.96
Cu(CH_3_COO)_2_	0.088	0.028–0.252	0.90	0.092	0.035–0.231	0.93

Note: dpf = days postfertilization; EC_50_ = effective concentration of 50% embryos hatching; CL = confidence limit.

## Data Availability

Not applicable.

## Supplementary Materials

The following supporting information can be downloaded at https://www.mdpi.com/article/10.3390/nano13192629/s1. Figure S1: Transmission electron microscopy (TEM) images of PEG@Cu NCs (A) and Kocide^®^ 3000 (B); Figure S2: Hydrodynamic particle size distribution of Kocide^®^ 3000; Table S1: The 96 hpf mortality of zebrafish embryo exposed to PEG@Cu NCs, Kocide^®^ 3000 and Cu(CH_3_COO)_2_.

## References

[1] Comber S., Deviller G., Wilson I., Peters A., Merrington G., Borrelli P., Baken S. (2023). Sources of Copper into the European Aquatic Environment. Integr. Environ. Assess. Manag..

[2] Nagakubo T., Kumano T., Ohta T., Hashimoto Y., Kobayashi M. (2019). Copper Amine Oxidases Catalyze the Oxidative Deamination and Hydrolysis of Cyclic Imines. Nat. Commun..

[3] Stern B.R., Solioz M., Krewski D., Aggett P., Aw T.-C., Baker S., Crump K., Dourson M., Haber L., Hertzberg R. (2007). Copper and Human Health: Biochemistry, Genetics, and Strategies for Modeling Dose-Response Relationships. J. Toxicol. Environ. Health Part B.

[4] Joachim S., Roussel H., Bonzom J.-M., Thybaud E., Mebane C.A., Van Den Brink P., Gauthier L. (2017). A Long-Term Copper Exposure in a Freshwater Ecosystem Using Lotic Mesocosms: Invertebrate Community Responses: Effects of Copper on Aquatic Invertebrates. Environ. Toxicol. Chem..

[5] Giampietro R., Spinelli F., Contino M., Colabufo N.A. (2018). The Pivotal Role of Copper in Neurodegeneration: A New Strategy for the Therapy of Neurodegenerative Disorders. Mol. Pharm..

[6] Liu X., Zhang J., Li J., Song C., Shi Y. (2022). Pharmacological Inhibition of ALCAT1 Mitigates Amyotrophic Lateral Sclerosis by Attenuating SOD1 Protein Aggregation. Mol. Metab..

[7] Malhotra N., Ger T.-R., Uapipatanakul B., Huang J.-C., Chen K.H.-C., Hsiao C.-D. (2020). Review of Copper and Copper Nanoparticle Toxicity in Fish. Nanomaterials.

[8] Pilehvar A., Town R.M., Blust R. (2020). The Effect of Copper on Behaviour, Memory, and Associative Learning Ability of Zebrafish (*Danio rerio*). Ecotoxicol. Environ. Saf..

[9] Aksakal F.I., Arslan H. (2020). Detoxification and Reproductive System-Related Gene Expression Following Exposure to Cu(OH)_2_ Nanopesticide in Water Flea (*Daphnia magna* Straus 1820). Environ. Sci. Pollut. Res..

[10] Becker D., Beckerman A.P. (2022). Copper Mediates Life History Responses of *Daphnia Pulex* to Predation Threat. Front. Ecol. Evol..

[11] Wan J.-K., Chu W.-L., Kok Y.-Y., Cheong K.-W. (2018). Assessing the Toxicity of Copper Oxide Nanoparticles and Copper Sulfate in a Tropical *Chlorella*. J. Appl. Phycol..

[12] Zhang C., Chen X., Tan L., Wang J. (2018). Combined Toxicities of Copper Nanoparticles with Carbon Nanotubes on Marine Microalgae *Skeletonema costatum*. Environ. Sci. Pollut. Res..

[13] Lamichhane J.R., Osdaghi E., Behlau F., Köhl J., Jones J.B., Aubertot J.-N. (2018). Thirteen Decades of Antimicrobial Copper Compounds Applied in Agriculture. A Review. Agron. Sustain. Dev..

[14] (2018). Final Renewal Report for the Active Substances Copper Compounds Finalised in the Standing Committee on Plants, Animals, Food and Feed at Its Meeting on 27 November 2018 in View of the Renewal of the Approval of the Active Substances Copper Compounds, as Candidate for Substitution, in Accordance with Regulation (EC) No 1107/20091.

[15] ICAMA http://www.chinapesticide.org.cn/zgnyxxw/zwb/dataCenter?hash=reg-info.

[16] Vignardi C.P., Muller E.B., Tran K., Couture J.L., Means J.C., Murray J.L.S., Ortiz C., Keller A.A., Smith Sanchez N., Lenihan H.S. (2020). Conventional and Nano-Copper Pesticides Are Equally Toxic to the Estuarine Amphipod *Leptocheirus plumulosus*. Aquat. Toxicol..

[17] Kah M., Beulke S., Tiede K., Hofmann T. (2013). Nanopesticides: State of Knowledge, Environmental Fate, and Exposure Modeling. Crit. Rev. Environ. Sci. Technol..

[18] Villaverde J.J., Sevilla-Morán B., López-Goti C., Alonso-Prados J.L., Sandín-España P. (2018). Considerations of Nano-QSAR/QSPR Models for Nanopesticide Risk Assessment within the European Legislative Framework. Sci. Total Environ..

[19] Mesquita A.F., Gonçalves F.J.M., Gonçalves A.M.M. (2023). The Lethal and Sub-Lethal Effects of Fluorinated and Copper-Based Pesticides—A Review. IJERPH.

[20] (1996). Water Quality—Determination of the Acute Lethal Toxicity of Substances to a Freshwater Fish [*Brachydanio rerio* Hamiltone-Buchanan (Teleostei, Cyprinidae)]—Part 2: Semi-Static Method.

[21] Yuan Z., Ma C., Jia M., Qian K. (2023). An Eco-Friendly Synthetic Polyethylene Glycol-Coated Copper Nanoclusters for Efficiently Suppress *Alternaria alternata* in Tobacco and Safety Evaluation. Ind. Crop. Prod..

[22] OECD (2013). Test No. 236: Fish Embryo Acute Toxicity (FET) Test.

[23] OECD (1998). Test No. 212: Fish, Short-Term Toxicity Test on Embryo and Sac-Fry Stages.

[24] De La Paz J., Beiza N., Paredes-Zúñiga S., Hoare M., Allende M. (2017). Triazole Fungicides Inhibit Zebrafish Hatching by Blocking the Secretory Function of Hatching Gland Cells. IJMS.

[25] Johnson A., Carew E., Sloman K. (2007). The Effects of Copper on the Morphological and Functional Development of Zebrafish Embryos. Aquat. Toxicol..

[26] Aksakal F.I., Sisman T. (2020). Developmental Toxicity Induced by Cu(OH)_2_ Nanopesticide in Zebrafish Embryos. Environ. Toxicol..

[27] Muller E.B., Lin S., Nisbet R.M. (2015). Quantitative Adverse Outcome Pathway Analysis of Hatching in Zebrafish with CuO Nanoparticles. Environ. Sci. Technol..

[28] Zhang Y., Zhang R., Sun H., Chen Q., Yu X., Zhang T., Yi M., Liu J.-X. (2018). Copper Inhibits Hatching of Fish Embryos via Inducing Reactive Oxygen Species and Down-Regulating Wnt Signaling. Aquat. Toxicol..

[29] Lee K.J., Nallathamby P.D., Browning L.M., Osgood C.J., Xu X.-H.N. (2007). In Vivo Imaging of Transport and Biocompatibility of Single Silver Nanoparticles in Early Development of Zebrafish Embryos. ACS Nano.

[30] Cheng J.P., Flahaut E., Cheng S.H. (2007). Effect of Carbon Nanotubes on Developing Zebrafish (*Danio rerio*) Embryos. Environ. Toxicol. Chem..

[31] Ghobadian M., Nabiuni M., Parivar K., Fathi M., Pazooki J. (2015). Toxic Effects of Magnesium Oxide Nanoparticles on Early Developmental and Larval Stages of Zebrafish (*Danio rerio*). Ecotoxicol. Environ. Saf..

[32] Santos D., Félix L., Luzio A., Parra S., Cabecinha E., Bellas J., Monteiro S.M. (2020). Toxicological Effects Induced on Early Life Stages of Zebrafish (*Danio rerio*) after an Acute Exposure to Microplastics Alone or Co-Exposed with Copper. Chemosphere.

[33] Klüver N., König M., Ortmann J., Massei R., Paschke A., Kühne R., Scholz S. (2015). Fish Embryo Toxicity Test: Identification of Compounds with Weak Toxicity and Analysis of Behavioral Effects To Improve Prediction of Acute Toxicity for Neurotoxic Compounds. Environ. Sci. Technol..

[34] Wang H., Zhou L., Liao X., Meng Z., Xiao J., Li F., Zhang S., Cao Z., Lu H. (2019). Toxic Effects of Oxine-Copper on Development and Behavior in the Embryo-Larval Stages of Zebrafish. Aquat. Toxicol..

[35] Brustein E., Saint-Amant L., Buss R.R., Chong M., McDearmid J.R., Drapeau P. (2003). Steps during the Development of the Zebrafish Locomotor Network. J. Physiol..

[36] González-Fraga J., Dipp-Alvarez V., Bardullas U. (2019). Quantification of Spontaneous Tail Movement in Zebrafish Embryos Using a Novel Open-Source MATLAB Application. Zebrafish.

[37] Aksakal F.I., Ciltas A. (2019). Impact of Copper Oxide Nanoparticles (CuO NPs) Exposure on Embryo Development and Expression of Genes Related to the Innate Immune System of Zebrafish (*Danio rerio*). Comp. Biochem. Physiol. Part C Toxicol. Pharmacol..

[38] Sun G., Liu K. (2017). Developmental Toxicity and Cardiac Effects of Butyl Benzyl Phthalate in Zebrafish Embryos. Aquat. Toxicol..

[39] Santos D., Luzio A., Matos C., Bellas J., Monteiro S.M., Félix L. (2021). Microplastics Alone or Co-Exposed with Copper Induce Neurotoxicity and Behavioral Alterations on Zebrafish Larvae after a Subchronic Exposure. Aquat. Toxicol..

[40] Kane A.S., Salierno J.D., Brewer S.K. (2005). Fish Models in Behavioral Toxicology: Automated Techniques, Updates and Perspectives. Methods in Aquatic Toxicology.

[41] Tierney K.B. (2011). Behavioural Assessments of Neurotoxic Effects and Neurodegeneration in Zebrafish. Biochim. Biophys. Acta (BBA)—Mol. Basis Dis..

[42] Li Y., Sun Y., Zhang G., He Z., Wang Y., Cui J. (2016). Effects of Copper Oxide Nanoparticles on Developing Zebrafish Embryos and Larvae. IJN.

[43] Zhang T., Xu L., Wu J.-J., Wang W.-M., Mei J., Ma X.-F., Liu J.-X. (2015). Transcriptional Responses and Mechanisms of Copper-Induced Dysfunctional Locomotor Behavior in Zebrafish Embryos. Toxicol. Sci..

