# Supplementary material for: Toxic Effects of Copper Fungicides on the Development and Behavior of Zebrafish in Early-Life Stages
Source: Nanomaterials (Basel). 2023 Sep 23;13(19):2629. doi: 10.3390/nano13192629 (PMC10574507; doi:10.3390/nano13192629)
Supplement: Supplementary file 1 [file nanomaterials-13-02629-s001.zip › nanomaterials-2573758-supplementary.pdf]

Supplementary material

Table S1. The 96 hpf mortality of zebrafish embryo exposed to PEG@Cu NCs, Kocide® 3000 and $Cu(CH_3COO)_2$.

PEG@Cu NCs		Kocide® 3000		$Cu(CH_3COO)_2$	
Concentration (mg/L)	Mortality (%)	Concentration (mg/L)	Mortality (%)	Concentration (mg/L)	Mortality (%)
Control	0.00	Control	6.41	Control	6.98
0.0125	2.22	0.25	6.98	0.23	19.51
0.05	31.11	0.5	4.44	0.26	29.27
0.2	53.33	1	7.78	0.304	65.85
0.8	84.45	2	6.67	0.349	94.87
3.2	93.33	4	16.67	0.402	100.00

Note: hpf = hours postfertilization.

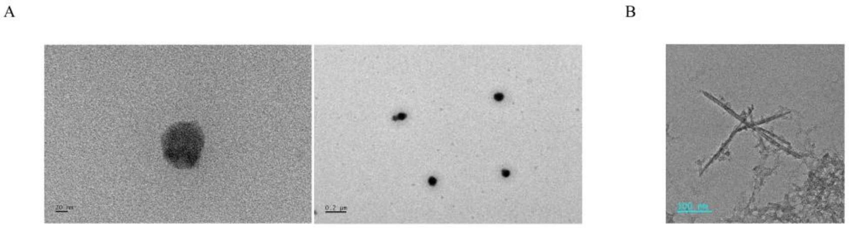

Figure S1. Transmission electron microscopy (TEM) images of PEG@Cu NCs (A) and Kocide® 3000 (B).

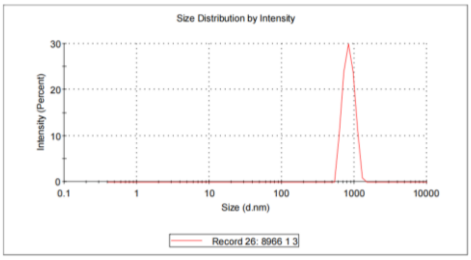

Figure S2. Hydrodynamic particle size distribution of Kocide® 3000.
